# Development of Acute Inflammatory Demyelinating Polyneuropathy 11 Days after Spinal Surgery: A Case Report and Literature Review

**DOI:** 10.1155/2021/6283076

**Published:** 2021-07-27

**Authors:** Eiichi Kakehi, Tadataka Kawakami, Yukiko Ishikawa, Takashi Matsuoka, Naoki Nakagawa, Tugutake Morishita, Shohei Taniguchi, Yukinobu Akamatsu, Shigehisa Sakurai, Akane Hirotani, Takafumi Nozaki, Keisuke Shoji, Seiji Adachi, Kazuhiko Kotani, Masami Matsumura

**Affiliations:** ^1^Department of General Medicine, Tottori Municipal Hospital, Tottori-City, Tottori, Japan; ^2^Department of Neurology, Shin-Oyama City Hospital, Oyama-City, Tochigi, Japan; ^3^Center for Community Medicine, Jichi Medical University, Shimotsuke-City, Tochigi, Japan; ^4^Department of Neurology, Tottori Municipal Hospital, Tottori-City, Tottori, Japan; ^5^Department of Neurological Surgery, Tottori Municipal Hospital, Tottori-City, Tottori, Japan

## Abstract

Guillain–Barré syndrome (GBS) usually has a good prognosis; however, patients may develop sequelae without prompt treatment. We herein describe an 81-year-old woman who developed acute-onset excruciating thigh pain and weakness in her lower extremities after spinal surgery. We diagnosed acute inflammatory demyelinating polyradiculoneuropathy by a nerve conduction study, which showed findings of demyelination without cerebrospinal fluid analysis because of a spinal prosthesis. Although anti-GM1 and anti-GalNAc-GD1a antibodies were positive, the patient was clinically diagnosed with acute inflammatory demyelinating polyradiculoneuropathy (a subtype of GBS), not acute motor axonal neuropathy. She recovered well with immunoglobulin therapy. A literature review of 18 cases revealed that unexplained weakness, areflexia, and numbness of the extremities after spinal surgery, a shorter time from spinal surgery to symptom onset to general GBS, abnormal nerve conduction study results, normal spinal imaging findings, and the development of atypical symptoms such as cranial and autonomic nerve syndrome and respiratory failure are useful for diagnosing GBS when cerebrospinal fluid examination cannot be performed after spinal surgery.

## 1. Introduction

Guillain–Barré syndrome (GBS) is an acute autoimmune inflammatory polyneuropathy that affects the peripheral nervous system and is usually triggered by infectious disease [1]. The incidence of GBS in the general population of the United States is reportedly 1-2 per 100,000 [2]. The postsurgical incidence of GBS is 4.1 per 100,000 patients, and the incidence after spinal surgery is 1 per 2,000 patients [3]. The onset of complications after spinal surgery ranges from 4% to 19% [4], and the overall incidence of neurologic complications is <1% [5]. The mortality rate of GBS within 1 year after diagnosis is reportedly 4.4% [6], and the rate of requiring mechanical ventilation is 38% [7].

The early symptoms of GBS mimic an acute spinal cord compression disorder or nerve root compression syndrome, and GBS can thus be difficult to differentiate from complications after spinal surgery. Cerebrospinal fluid (CSF) examination is essential for diagnosing GBS; however, postoperative spinal changes often make CSF collection and analysis difficult.

Although GBS is a rare complication after spinal surgery, its correct diagnosis is crucial because it will prevent a reoperation based on a misdiagnosis of recurrent spinal problems. We herein describe a case of GBS that mimicked spinal cord injury after spinal surgery. Although the characteristics of GBS after spinal surgery have been previously reported [8], the strategies for differentiating postoperative complications and GBS have not been well summarized. We reviewed previous reports to clarify these diagnostic challenges.

## 2. Case Presentation

**Figure 1:**
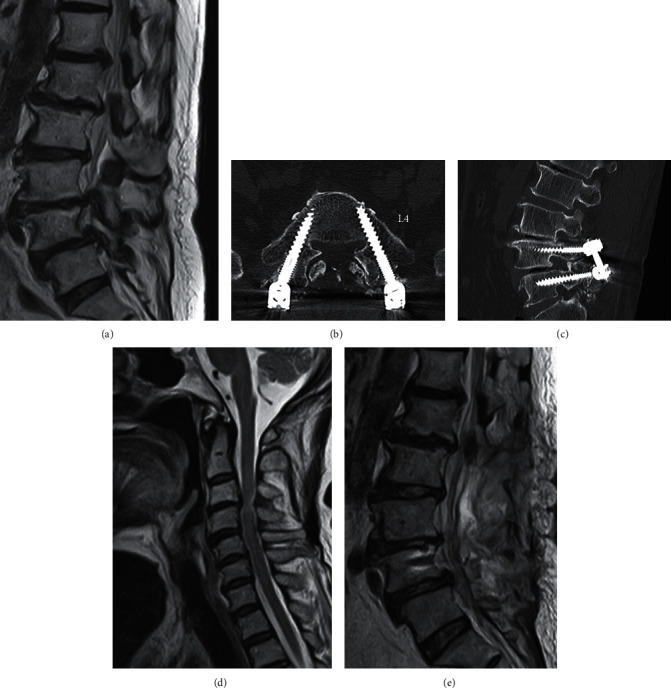
Imaging findings. (a) Lumbar magnetic resonance imaging (MRI) demonstrating severe lumbar spine stenosis at L1–5 and spondylolisthesis at L4/5. (b), (c) No percutaneous pedicle screw deviation or compression observed in the spinal canal. (d) Cervical MRI revealing spinal canal stenosis extending to C4–6 and disc herniation at the C3/4 level. (e) Lumbar MRI showing bilateral foraminal narrowing at the L4/5 level after laminectomy for L4 anterior spondylolysis, but no apparent implant deviation.

An 81-year-old Japanese woman with a history of cervical canal stenosis and bilateral knee osteoarthritis visited our outpatient orthopedics department with the chief complaint of claudication and pain in the back of the thigh. These symptoms had been present for 1 year and had worsened 3 months before her visit. She had developed no recent infections nor received any vaccinations in the previous few months, and she had no surgical history. Muscle strength testing according to the Medical Research Council scale demonstrated grade 5/5 muscle strength in the bilateral upper and lower extremities, but her bilateral extensor digitorum longus and brevis strength was 4/5. Her bilateral patellar and Achilles tendon reflexes were brisk. On sensory examination, she showed hyperesthesia in the bilateral distal part of lower legs on light touch testing. Magnetic resonance imaging (MRI) showed severe lumbar spinal stenosis at L1–5 and spondylolisthesis at L4/5 (Figure 1(a)). She was diagnosed with spondylolisthesis with lumbar spinal stenosis. An L1–5 laminectomy and L4/5 posterolateral fusion were performed under general anesthesia without surgical complications. Her claudication improved, and she was able to ambulate with a walker. She developed no acute complications for 8 days after the surgery.

Nine days after the surgery, she developed only one episode of transient soft stool without fever, abdominal pain, nausea, or vomiting. Eleven days after the surgery, she reported acute onset of excruciating bilateral thigh pain. She subsequently developed difficulty walking because of ascending weakness of her bilateral lower extremities. The tingling in her bilateral lower extremities was exacerbated. On physical examination, she was conscious and afebrile, and her surgical wound was healing well. All cranial nerves were intact, and she had no difficulty swallowing or breathing. Muscle strength testing showed grade 4/5 in the upper distal extremities and 3/5 in the lower distal and proximal extremities, with no muscle atrophy. On sensory examination, the hyperesthesia in the bilateral distal parts of her lower legs was exacerbated on light touch testing. The bilateral tendon reflexes of the biceps brachii, triceps brachii, and brachioradialis were normal, but the bilateral patellar and Achilles tendon reflexes were absent. Pathological reflexes, including those in the upper and lower extremities, were negative. She was unable to stand or walk. Routine blood tests, including a complete blood count, glucose, electrolytes, renal function, and liver enzymes, were within normal limits. Serum anti-GM1-IgG and anti-GalNAc-GD1a-IgG antibodies were positive, with optical density values (control <0.1) of 0.157 and 0.135, respectively.

**Figure 2:**
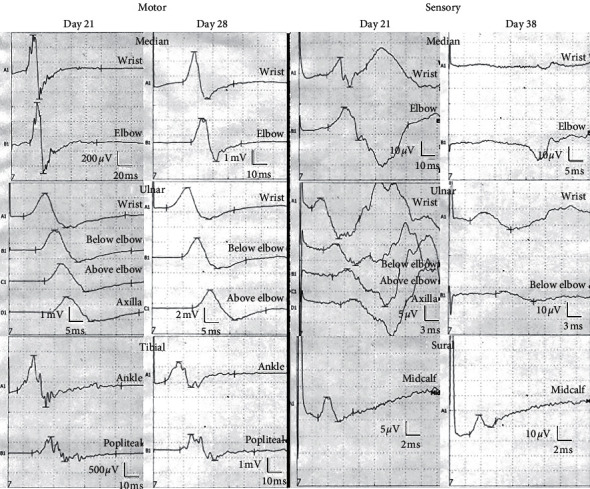
Nerve conduction study results. At 21 days after surgery, the median and tibial motor nerves showed findings of demyelination, such as prolonged terminal latencies, reduction in the compound muscle action potential, a slowed conduction velocity, and temporal dispersion. The ulnar motor nerve showed a decrease in amplitude. The sensory examination showed an abnormal median and normal sural sensory response. The F wave was absent in the bilateral median, ulnar, and tibial motor nerves (data not shown). At 38 days after surgery, the median and ulnar motor nerve compound muscle action potentials had residual prolonged terminal latencies but improved amplitudes. The tibial nerve showed temporal dispersion on both day 21 and day 28 with no improvement. The median and ulnar sensory nerves abnormalities were exacerbated, but the sural nerve remained normal.

Spinal computed tomography showed no evidence of spinal cord compression (Figures 1(b) and 1(c)). Brain MRI showed no abnormalities. MRI of the whole spine showed moderate disc herniation at the C3/4 level without significant cord or nerve root compression or an epidural hematoma or abscess (Figures 1(d) and 1(e)). An acute polyradiculoneuropathy was suspected based on rapidly progressive ascending weakness, sensory disturbance, and areflexia of the lower extremities without implant-related complications. We therefore consulted a neurologist, and a nerve conduction study (NCS) was performed 21 days after surgery.  Table 1 and Figure 2 show the results of the NCS. The median and tibial motor nerves showed findings of demyelination [9], such as prolonged terminal latencies, reduction in the compound muscle action potential, a slowed conduction velocity, and temporal dispersion. The ulnar motor nerve showed a decrease in amplitude. The F wave was absent in the bilateral median, ulnar, and tibial motor nerves. The sensory examination showed an abnormal median and normal sural sensory response [10]. Because we did not perform needle electromyography, therefore, we could not evaluate fibrillation potentials. In addition, we did not identify visible fasciculation in any involved muscles or any new central nerve disorders using neuroimaging studies. A CSF examination was difficult to perform because the patient had recently undergone lumbar spinal surgery. Given the acute onset of her symptoms, normal spinal imaging findings, and significant evidence of demyelination on the NCS, she was diagnosed with GBS.

Treatment was started 9 days after the appearance of symptoms. Immunoglobulin therapy was started at a dose of 0.4 g/kg for 5 days. After 1 month of follow-up, her upper and lower extremity weakness resolved, and motor examination showed grade 5/5 in the upper extremities and 4/5 in the lower extremities. She had residual tingling in both lower extremities. An NCS performed 38 days after surgery showed that the median and ulnar motor nerve compound muscle action potentials had residual prolonged terminal latencies but improved amplitudes. There was no improvement in the tibial nerve with temporal dispersion on day 21 and day 38. The median and ulnar sensory nerve abnormalities were exacerbated, but the sural nerve remained normal (Table 1, Figure 2). She needed a cane to walk, but muscle strength testing according to the Medical Research Council scale showed that her extremity muscle strength had improved to grade 5/5. She was able to ambulate with a cane and was subsequently transferred to a rehabilitation hospital.

## 3. Literature Review

To understand the clinical characteristics of our case, we reviewed case reports of GBS after spinal surgery. A search of the PubMed database was performed using the terms “Guillain–Barré syndrome” AND “spinal surgery OR spine surgery” for all articles published until May 2021. We identified 14 published reports involving 18 patients with GBS after spinal surgery (Table 2) [11–24].

Table 2 summarizes the characteristics of previously described patients diagnosed with GBS after spinal surgery. Their average age was 56.8 years (range, 33–73 years), and the male/female ratio was 11/7 [11–24]. The vertebral regions affected by the surgery were the lumbar region in eight (44.4%) patients [16, 17, 20–24], the cervical region in four (22.2%) [15,18], the thoracolumbar region in three (16.7%) [11, 14, 18], the thoracic region in one (5.6%) [13], the thoracosacral region in one (5.6%) [11], and the lumbosacral region in one (5.6%) [19]. The average duration of time from spinal surgery to the onset of GBS symptoms was 7.4 days (range, 1 hour to 22 days), with two patients having a duration of ≤3 hours [17], and the duration of time from onset of GBS symptoms to diagnosis was 7.2 days (range, 1–19 days). Ten (55.6%) patients had motor weakness at the nadir in the lower limbs only [11, 15, 17–20, 22–24], and eight (44.4%) had motor weakness in the upper and lower limbs [12–14, 16–18, 21]. Seven (38.9%) patients had sensory deficits at the nadir in the lower limbs only [11, 16, 17, 20, 22–24], five (27.8%) in the upper and lower limbs [12, 13, 15, 17, 19], and one (11.1%) in the upper limbs only [14]. Eleven (61.1%) patients showed areflexia or hyporeflexia [11–17, 20–23]. Three (16.7%) patients reported pain [17, 22, 24]. Nine (50.0%) patients had cranial nerve symptoms [13, 14, 16, 18, 19, 23, 24], which included facial paralysis, dysphagia, decreased gag reflex, and impaired vocalization. Five (27.8%) patients had autonomic nerve symptoms [13–15, 19, 23], which included fever, tachycardia, atrial fibrillation, urinary retention, abdominal pain, diarrhea, and constipation. Nine (50.0%) patients needed mechanical ventilator support by intubation [11, 13, 14, 17–20], one patient could not be weaned [18], and the rest received temporary support. Seven (38.9%) patients needed a reoperation to determine the cause of postoperative complications [14, 15, 17, 18]. CSF examinations were performed in 10 (55.6%) patients, 6 of 12 (50.0%) patients who underwent lumbar surgery [12, 16, 17, 21, 23], and 4 of 5 (80.0%) patients who underwent nonlumbar surgery [13, 15, 18]). Fifteen (88.2%) patients underwent an NCS [11–20,22–24]. Antiganglioside antibodies were detected in two patients [15, 17], but the prior infection status was unknown. Seventeen patients underwent intravenous immunoglobulin (IVIg) therapy [12–24], three patients received concomitant plasma exchange (PE) therapy [12, 17], and two patients received concomitant high-dose corticosteroids [12, 23]. One patient received high-dose corticosteroids only [11]. No deaths occurred. The motor impairment was improved in many patients; however, some had residual sensory disturbances.

## 4. Discussion

**Figure 3:**
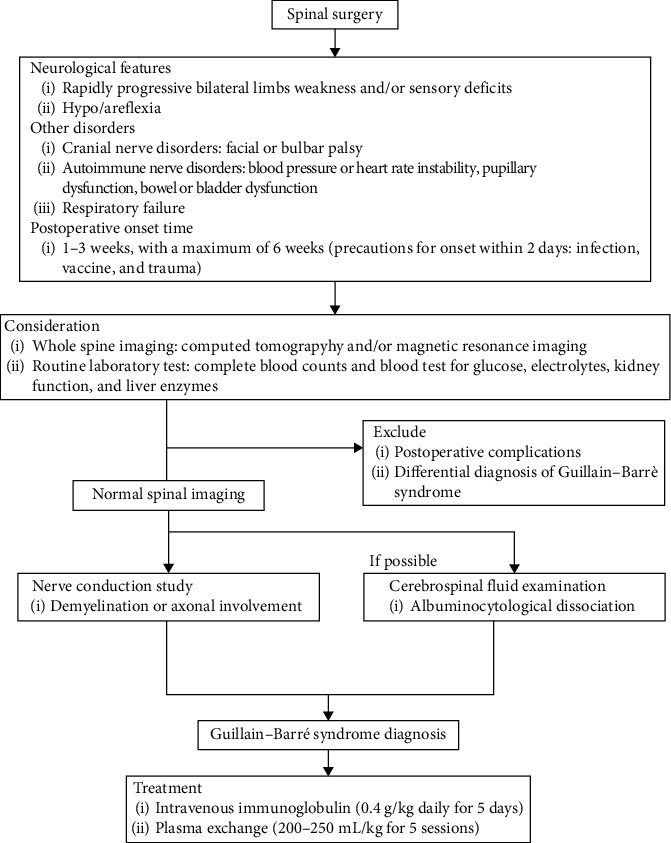
Strategies for diagnosis and treatment of Guillain–Barré syndrome after spinal surgery.

Clinicians should be aware of progressive muscle weakness, areflexia, or numbness after a spinal operation as potential signs of GBS. The clinical features that are helpful for the diagnose of GBS after spinal surgery can be summarized from previous literature as follows: shorter time from spinal surgery to symptom onset to general GBS, abnormal NCS results, normal spinal imaging, and the development of atypical symptoms such as cranial and autonomic nerve syndrome and respiratory failure. We consider that even when CSF testing is not available, an NCS is preferable to CSF analysis in the diagnosis of GBS and is clinically useful. Furthermore, rapid initiation of treatment with IVIg and/or PE is useful for improving the prognosis [7]. GBS after spinal surgery presents a unique diagnostic challenge, and we devised a flowchart (Figure 3) based on previous literature.

The mechanism of GBS development most likely involves a recent preceding upper or lower respiratory tract infection or gastrointestinal illness followed by an immunological response [25]. The mechanism by which GBS develops after surgery is considered to involve an interaction between the anesthetic agents and peripheral nerve myelin or local trauma to roots; this may initiate a cascade of immunologic events that result in demyelinating neuropathy [26]. Staff et al. [27] reported an inflammatory change in nerve biopsy samples from patients with nontraumatic postoperative neuropathy within 30 days after surgery. Surgery alters the balance of the immune system and causes transient immunosuppression, which can trigger the onset of GBS and promote subclinical infection that can also predispose a patient to GBS [28, 29].

Serum anti-GM1 and anti-GalNAc-GD1a antibodies, which were detected in our case, are reportedly associated with acute motor axonal neuropathy in electrophysiological studies due to preinfection by *Campylobacter jejuni* [30, 31]. Ogawa et al. [32] reported that the presence of anti-GM1/GalNAc-GD1a antibodies was correlated with pure motor GBS characterized by antecedent respiratory infection, fewer cranial nerve deficits, and conduction blocks at intermediate sites of motor nerves. We did not perform serological testing for *C. jejuni* because our patient had only one episode of soft stool, had not ingested any components that could cause infectious enteritis, and was not considered to have *Campylobacter* enteritis. Therefore, the relationship between the antiganglioside antibodies and the patient's symptoms was unclear.

Most reported cases of postoperative GBS occurred within 1–3 weeks, with a maximum of 6 weeks [3]. Our literature review indicated that the onset of GBS symptoms after spinal surgery tended to be earlier than that after nonspinal surgery, although it was later than the occurrence of other complications such as iatrogenic spinal cord injury or postoperative herniation [19]. However, Wakerley and Yuki [33] reported that the onset of GBS within 2 days after surgery would be triggered not by the surgery but by preoperative triggers and subsequent immune mechanisms (e.g., infection, vaccines, and trauma). Xu et al. [24] reported that a patient developed GBS 2 days after surgery and had received an influenza vaccine 10 days before surgery. The duration of postoperative symptom onset and a careful preoperative history can help to reveal the cause.

An NCS is the most useful examination for GBS after spinal surgery because an NCS is less likely to be affected by the postoperative state than is a CSF analysis [19]. An NCS in patients with GBS typically shows a multifocal demyelinating process, including conduction block or temporal dispersion in motor nerves [25]. Abnormal median and normal sural sensory responses are highly suggestive of acute inflammatory demyelinating polyneuropathy (AIDP) subtypes of GBS [10]. Our patient exhibited these findings, which were useful for diagnosing the underlying cause of her muscle weakness. Uncini and Kuwabara proposed the characteristics of “nodopathy” as rapidly reversible nerve conduction block due to paranodal myelin detachment and nodal sodium channel disruption, the absence of excessive temporal dispersion associated with remyelination, and eventual complement-mediated axonal degeneration depending on the severity [34]. In the present case, the findings of temporal dispersion of the tibial nerve at day 38 (after the symptoms had improved) and no evidence of conduction block support the diagnosis of AIDP rather than nodopathy. Anti-GM1 antibodies have been reported to be associated with acute motor axonal neuropathy, but our literature review revealed an association between acute motor and sensory axonal neuropathy (AMSAN) [15] and AIDP [17]. Our literature review also showed that AMSAN has recently been associated with spinal surgery [13, 15, 22]. AMSAN is rare and usually presents with severe symptoms over a short period, and patients often experience prolonged and incomplete recovery compared with other forms of GBS. The maximum incidence of electrophysiological abnormalities is reportedly 4–12 weeks after the onset of neurological symptoms [35]; therefore, the worsening of ulnar nerve sensory deficits in this case is presumed to be a clinical process. Because the NCS findings are not confirmed until 5 days after the onset of symptoms, a repeated NCS can help diagnose GBS [36].

The absence of abnormal findings in spinal imaging is also useful for diagnosing GBS. The differential diagnoses of complications after spinal surgery often include spinal cord or root nerve injury during positioning, hemorrhage, recurrent disc herniation, infection, displacement of an implant, vascular insults (spinal cord ischemia/injury), or pharmacological toxicity, with a reported complication rate of 4–19% [4,5]. In our review, reoperation was performed in seven (38.9%) patients [14, 15, 17, 18, 21, 22]. Space-occupying lesions, such as an extradural hematoma or displaced operative implant, can be improved after a reoperation; however, complications might be worsened by an inappropriate operation when the origin of impairment is unclear [18]. When neurological deficits are observed after spinal surgery, GBS should be considered as a differential diagnosis when no abnormalities are present in the spine.

Although the finding of albuminocytologic dissociation in a CSF examination is helpful for the diagnosis of GBS, CSF examination is often difficult to perform after spinal surgery because of spinal changes, spinal hardware, wounds, or postoperative contamination. In this literature review, the rate of CSF examination after lumbar surgery was lower than that after cervical and thoracic vertebral surgery. The protein levels in CSF may be normal in early GBS, but they are elevated in 90% of patients by the end of the second week of symptoms [37]. Albuminocytologic dissociation also occurs with compression of the spinal cord [38]. This review suggests that the onset of postspinal surgery GBS is earlier than that of general GBS and that CSF testing may be performed early in the disease course. Therefore, normal or elevated CSF protein levels cannot be used to rule out or confirm a diagnosis of GBS. However, the absence of pleocytosis in CSF helps to differentiate GBS from other infectious, inflammatory, and malignant diseases [39]. If possible, CSF examination should be performed not only for the diagnosis of GBS but also for other differential diagnoses.

Our review of the literature also revealed cranial nerve involvement, autonomic dysfunction, and respiratory disorders as clinical features of GBS after spinal surgery. In this review, cranial nerve involvement was found in 50% of cases and was more common in GBS after spinal surgery than in general GBS [40]. Umer et al. [41] reported that if facial, bulbar, and neck weakness progresses within 5 days, impending respiratory failure can be expected. Autonomic dysfunction such as cardiac arrhythmia, hypertension, hypotension, diarrhea, and ileus might occur in patients with GBS [42], and the presence of these signs can trigger clinicians' recognition of GBS. In this review, gastrointestinal symptoms were the most common; in particular, diarrhea must be differentiated from infectious enteritis. This review also showed that mechanical ventilator management was needed in 50% of cases and was more frequent than in patients with general GBS [7]. Clinicians should consider the appearance of cranial neurological, autonomic, and respiratory symptoms after spinal surgery.

Prompt administration of IVIg or PE is essential for neurologic recovery in patients with GBS. These treatments should ideally be started as soon as possible; a milder benefit has been shown with treatment that is started up to 4 weeks after symptom onset [43]. Eight randomized controlled trials of the efficacy of corticosteroids for GBS showed no benefit, and treatment with oral corticosteroids was even shown to have a negative effect on outcomes [44]. In this review, only one patient required persistent ventilator management and did not improve; although the remaining patients improved, some had sequelae of weakness, numbness, facial paralysis, and dysphagia. Because no clear treatment strategy for GBS after spinal surgery has been reported, we herein propose a diagnostic flowchart for this disease (Figure 3).

## 5. Conclusion

GBS after spinal surgery is a rare neurologic complication that presents unique diagnostic challenges. Prompt diagnosis and appropriate treatment will prevent GBS from progressing to a life-threatening status. When patients exhibit unexplained weakness, areflexia, and numbness of the extremities 1–3 weeks after spinal surgery, the absence of abnormal findings in spinal imaging should be confirmed first. In patients without postoperative spinal changes, an NCS has priority regardless of whether CSF testing is available.

## Figures and Tables

**Table 1:** Electrophysiological findings 21 and 38 days after spinal surgery.

	Site	Terminal latency (ms), motor: median <3.7, ulnar <2.9, tibial <5.1, sensory: median <2.9, ulnar <2.6, sural <3.1				Amplitude (motor = mV, sensory = *µ*V), motor: median >4.9, ulnar >5.5, tibial >10.1, sensory: median and ulnar >29.4, sural >3.3				Conduction velocity (m/s), motor: median >58, ulnar >59, tibial >48, sensory: median >65, ulnar >64, sural >46						FCV (m/s), median >69								
		Day 21		Day 38		Day 21		Day 38		Day 21			Day 38			Day 21					Day 38			
		Right	Left	Right	Left	Right	Left	Right	Left	Right		Left	Right		Left	Right		Left			Right			Left
Motor																								
Median	Wrist	20.0	16.6	20.1	17.8	0.87	1.37	3.21	2.73	35.6		31.0	38.3		33.9	NR		NR		44.3			36.5	
Ulnar	Wrist	4.90	4.65	4.55	5.30	2.26	3.52	4.09	5.01	41.0		50.6	34.3		42.9	NR		NR		NR			NR	
Tibial	Ankle	6.10	9.60	7.80	11.20	1.70	0.83	1.65	0.95	34.5		27.2	30.1		34.4	NR		NR		36.9			48.7	

Sensory																								
Median	Wrist	NR	NR	NR	NR	NR	NR	NR	NR	NR	NR		NR	NR		—	—		—			—		
Ulnar	Wrist	2.46	2.40	2.66	2.58	13.20	16.50	10.70	13.10	67.5	51.3		53.1	53.9		—	—		—			—		
Sural	Midcalf	2.70	3.00	2.60	3.10	7.40	3.30	8.10	6.40	51.1	46.7		53.8	45.2		—	—		—			—		

FCV, F wave conduction velocity; NR, no response.

**Table 2:** Characteristics of previous patients diagnosed with Guillain–Barré syndrome after spinal surgery.

No.	Authors	Age/sex	Spinal surgery			Postoperative onset time	Diagnostic delay	Motor weakness at the nadir			Sensory deficits at the nadir				Tendon reflex		Pain		
1	Stambough et al. [11]	33/F	Arthrodesis (T5–L2)			20 days	4 days	Bilateral lower limbs			Perioral and distal lower limbs				Areflexia		NR		
2	Riebel et al. [12]	62/F	Arthrodesis (T11–S)			22 days	3 days	Bilateral upper and lower limbs (distal > proximal)			Bilateral upper and lower limbs (distal > proximal)				Areflexia		NR		
3	Cheng et al. [13]	59/F	Laminoplasty (T1–3), tumor resection			6 hours	8 days	Bilateral upper and lower limbs (distal > proximal)			Bilateral upper and lower limbs				Areflexia		NR		
4	Son et al. [14]	50/M	Spinal canal decompression fusion (T10–L2)			8 days	2 days	Bilateral upper and lower limbs			Bilateral hands				Areflexia		NR		
5	Miscusi et al. [15]	55/M	Laminoplasty (C6/7)			36 hours	NR	Bilateral lower limbs			Below the level of T5				Areflexia		NR		
6	Battaglia et al. [16]	73/F	Kyphoplasty (L1)			7 days	NR	Bilateral upper and lower limbs			Bilateral lower limbs				Areflexia		NR		
7	Boghani et al. [17]	58/M	Laminoplasty (L4/5)			3 hours	2 days	Bilateral upper and lower limbs			Ascended to upper thighs, then to the abdomen, thorax, and arms				Hyporeflexia		Yes		
8	—	40/M	Laminoplasty (L3/4)			1 hour	NR	Bilateral lower limbs			Bilateral lower limbs				NR		NR		
9	Huang et al. [18]	50/M	Occipitocervical fusion (occiput–C2), anterior cervical discectomy and fusion (C5/6)			7 days	16 days	Bilateral upper and lower limbs			NR				NR		NR		
10	—	53/M	Laminoplasty (C3–6)			3 days	19 days	Bilateral lower limbs			NR				NR		NR		
11	—	69/M	Instrumented posterior approach, lumbar interbody fusion (T10–L5)			2 days	12 days	Bilateral upper and lower limbs			NR				NR		NR		
12	—	58/M	Anterior cervical discectomy and fusion (C4–7)			3 days	4 days	Bilateral lower limbs			NR				NR		NR		
13	Chen et al. [19]	57/M	Spinal fusion (L3–S1)			5 days	6 days	Bilateral lower limbs			Below the level of T3				NR		NR		
14	Rashid et al. [20]	62/F	Spinal fusion (L2–4)			10 days	2 days	Bilateral lower limbs			Bilateral lower limbs				Areflexia		NR		
15	Sahai et al. [21]	52/M	Laminectomy (L4/5)			17 days	2 days	Bilateral lower limbs and left arm			NR				Areflexia		NR		
16	Dowling and Dowling [22]	53/F	Laminectomy (L1/2)			17 days	7 days	Bilateral lower limbs			Bilateral lower limbs				Areflexia		Yes		
17	Sanpei et al. [23]	70/F	Corrective surgery (L2/3)			5 days	14 days	Bilateral lower limbs			Bilateral lower limbs (distal > proximal)				Areflexia		NR		
18	Xu et al. [24]	70/M	Spinal fusion (L4/5)			2 days	NR	Bilateral lower limbs			Bilateral lower limbs (distal > proximal)				Normal		Yes		
—	—	Average 56.8	Lumbar spine	8 (44.4%)		Average	Average	Lower limbs only		10 (55.6%)	Lower limbs only			7 (38.9%)	11 (61.1%)		3 (16.7%)		
—	—	M/F ratio 11 : 7	Cervical spine	4 (22.2%)		7.4 days	7.2 days	Upper and lower limbs		8 (44.4%)	Upper and lower limbs			5 (27.8%)	—		—		
—	—	—	Thoracolumbar	3 (16.7%)		—	—	Upper limbs only		0 (0.0%)	Upper limbs only			1 (11.1%)	—		—		
—	—	—	Thoracic spine	1 (5.6%)		—	—	—		—	NR			5 (27.8%)	—		—		
—	—	—	Thoracosacral	1 (5.6%)		—	—	—		—	—			—	—		—		
—	—	—	Lumbosacral	1 (5.6%)		—	—	—		—	—			—	—		—		
19	Present case	81/F	Laminectomy (L1–5), posterolateral fusion (L4/5)			11 days	9 days	Bilateral lower limbs			Bilateral lower limbs				Areflexia		Yes		

No.	Autonomic nerve dysfunction		Ventilator support	Reoperation for exploration	CSF^†^		NCS	Antiganglioside antibody				Treatment	Follow-up (months)		Prognosis			
1	No		Yes	No	Missing		AIDP	No				High dose DEX	24		Neurologic deficits were improved.			
2	No		No	No	Yes		AIDP	No				High dose corticosteroid, IVIg, PE	6		The neurological deficits, except for minor weakness of the intrinsic muscles of the hands, had completely resolved.			
3	Fever, tachycardia, diarrhea		Yes	No	Yes		AMSAN	No				IVIg	7		Was able to transfer from bed to chair.			
4	Abdominal pain		Yes	Yes	Missing		AIDP	No				IVIg	2		The neurological deficits, except for minor weakness of the intrinsic muscles of the hands, had completely resolved.			
5	Fever, diarrhea		No	Yes	Yes		AMSAN	GM1				IVIg, mesalazine, meropenem	3		Progressive proximal-distal recovery of strength in both legs.			
6	No		No	No	Yes		AIDP	No				IVIg	4		Left peripheral facial palsy.			
7	No		Yes	Yes	Yes		AIDP	No				IVIg, PE	12		Paresthesia in lower trunk and legs.			
8	No		No	Yes	Yes		AIDP	GM1				IVIg, PE	18		Residual numbness of legs.			
9	No		Yes	Yes	Missing		Missing	No				IVIg	22		Was able to walk with a cane.			
10	No		Yes	No	3053/8		AIDP	No				IVIg	22		Was able to transfer from bed to chair.			
11	No		Yes	No	Missing		AIDP	No				IVIg	11		Ventilator support was required.			
12	No		No	No	500/5		AIDP	No				IVIg	7		Minor weakness of the intrinsic muscles of the hands.			
13	Atrial fibrillation, urinary retention, constipation		Yes	No	Missing		AIDP	No				IVIg	16		Left diaphragm weakness.			
14	No		Yes	No	Missing		AIDP	No				IVIg	12		Was able to ambulate independently.			
15	No		No	Yes	Yes		Missing	No				IVIg	6		Was able to ambulate without significant intervention.			
16	No		No	Yes	Missing		AMSAN	No				IVIg	2.5		Nearly recovered.			
17	Hypertension, constipation		No	No	1216/6		AIDP	No				IVIg, mPSL	NR		Was able to move her facial muscles and, gradually, her limbs.			
18	No		No	No	Missing		AIDP	No				IVIg	1		Regained 5/5 strength in extremities and could ambulate without aid.			
—	Dysphagia	Digestive symptom	5 (27.8%)	9 (50.0%)	7 (38.9%)		10 (55.6%)	Total	16 (88.9%)	2 (11.1%)		IVIg	17 (94.4%)		—	Improved		17 (94.4%)
—	Facial palsy	Fever	2 (11.1%)	—	—		—	AIDP	13 (72.2%)	—		PE	3 (16.7%)		—	Not improved		1 (5.6%)
—	Ptosis	Arrhythmia	2 (11.1%)	—	—		—	AMSAN	3 (16.7%)	—		IVIg + PE	3 (16.7%)		—	—		—
—	—	Urinary retention	1 (5.6%)	—	—		—	—	—	—		Steroid	3 (16.7%)		—	—		—
19	Diarrhea		No	No	Missing		AIDP	GM1, GalNAc-GD1a				IVIg	1		Was able to ambulate with a cane.			

F, female; M, male; NR, not reported; CSF, cerebrospinal fluid; NCS, nerve conduction study; AMSAN, acute motor and sensory axonal neuropathy; AIDP, acute inflammatory demyelinating polyneuropathy; DEX, dexamethasone; IVIg, intravenous immunoglobulin; PE, plasma exchange; mPSL, methylprednisolone. ^†^If albuminocytologic dissociation was present, it is shown as protein (mg/L)/white blood cell count.
